# EHMTI-0382. A distal epidural blood patch relieves low CSF pressure headache

**DOI:** 10.1186/1129-2377-15-S1-J7

**Published:** 2014-09-18

**Authors:** CJ Hung, CC Wu

**Affiliations:** 1 Anesthesiology, Taichung Veterans General Hospital, Taichung, Taiwan

## Introduction

Low CSF pressure headache is a clinical syndrome that identifies patients who were previously headache-free and who develop a persistent headache over 24 h, or just a few days. The diagnostic feature of low CSF pressure headache is that of worsening when upright and significant improvement on lying flat. Conservative treatment options include bed rest, abdominal binder, hydration, caffeine, and corticosteroids. If conservative treatment fails, epidural blood patching is often the next option.

## Aim

Since the accumulation of CSF leakage spreads widely, the injection site for the epidural blood patch may be difficult to choose in the management of low CSF pressure headache. We prefer the distal approach for the epidural blood patch which will be demonstrated in the following case.

## Case report

A 31-year-old man spontaneously developed orthostatic headaches for 2 weeks. The pachymeningeal enhancement of brain is noted with venous sinus engorgement in the images of brain MRI. Spinal MRI demonstrated CSF signals along bilateral C5-6, C6-7, C7-T1, T1-2, T8-9, T9-10, T10-11, and T11-12 neural foramens with epidural fluid accumulation from C7 to T12 compatible with CSF leakage. Conservative treatment with bed-rest, intravenous fluid infusion of 3000 ml QD, nonsteroidal anti-inflammatory drugs, and caffeine failed. An epidural blood patch of 6mL through T10-11 interspinous space resulted in immediate headache relief.

## Conclusion

Some patients have spontaneous low CSF pressure headache, most often due to a cryptic CSF leak. Epidural blood patch at distal part relieves headache by immediately compressing the dural sac and raising intrathecal pressure.

No conflict of interest.

